# COVID-19 Vaccine Acceptance, Attitude and Perception among Slum and Underserved Communities: A Systematic Review and Meta-Analysis

**DOI:** 10.3390/vaccines11050886

**Published:** 2023-04-23

**Authors:** Joseph Kawuki, Siyu Chen, Yuan Fang, Xue Liang, Paul Shing-fong Chan, Zixin Wang

**Affiliations:** 1 Jockey Club School of Public Health and Primary Care, The Chinese University of Hong Kong, Hong Kong, China; 2 Department of Health and Physical Education, The Education University of Hong Kong, Hong Kong, China

**Keywords:** slum, underserved communities, vaccine acceptance, vaccine hesitancy, COVID-19, attitude and perception, vaccine uptake

## Abstract

This systematic review summarises the literature on Coronavirus Disease 2019 (COVID-19) vaccination, including acceptance, uptake, hesitancy, attitude and perceptions among slum and underserved communities. Relevant studies were searched from PubMed, Scopus, Web of Science and Google Scholar, following a pre-registered protocol in PROSPERO (CRD42022355101) and PRISMA guidelines. We extracted data, used random-effects models to combine the vaccine acceptance, hesitancy and uptake rates categorically, and performed meta-regression by R software (version 4.2.1). Twenty-four studies with 30,323 participants met the inclusion criteria. The overall prevalence was 58% (95% CI: 49–67%) for vaccine acceptance, 23% (95% CI: 13–39%) for uptake and 29% (95% CI: 18–43%) for hesitancy. Acceptance and uptake were positively associated with various sociodemographic factors, including older age, higher education level, male gender, ethnicity/race (e.g., Whites vs African Americans), more knowledge and a higher level of awareness of vaccines, but some studies reported inconsistent results. Safety and efficacy concerns, low-risk perception, long distance to vaccination centres and unfavourable vaccination schedules were prominent reasons for hesitancy. Moreover, varying levels of attitudes and perceptions regarding COVID-19 vaccination were reported with existing misconceptions and negative beliefs, and these were strong predictors of vaccination. Infodemic management and continuous vaccine education are needed to address existing misconceptions and negative beliefs, and this should target young, less-educated women and ethnic minorities. Considering mobile vaccination units to vaccinate people at home or workplaces would be a useful strategy in addressing access barriers and increasing vaccine uptake.

## 1. Introduction

Since its emergency in late 2019, the Coronavirus Disease 2019 (COVID-19) remains a prioritised global health concern. As of 22 March 2023, over 682 million cases and 6.8 million deaths have been recorded worldwide [1]. The pandemic has caused profound socio-economic impacts in all countries, which are still evident [2]. This was mainly due to the restrictive measures, such as lockdowns, restricted movement and school closures, among others, adopted by different countries to minimise the rapid spread of the virus [2]. 

The rollout of COVID-19 vaccination programs contributed to the control of the pandemic and allowed several countries to lift strict control measures [3,4]. As of 18 January 2023, about 69.2% of the global population had received at least one dose of the COVID-19 vaccine, with over 13.2 billion doses given [5]. However, only 25.9% of people in low-income countries had received at least one dose [5], highlighting inequality in vaccination access and uptake [6]. Low-income countries have reported issues such as global vaccine supply chain dynamics, inaccessibility of vaccination centres and perceived misconceptions about the vaccine as prominent reasons for the low COVID-19 vaccine uptake [7,8,9]. Moreover, vaccine hesitancy, a phenomenon of “delayed acceptance or refusal of safe vaccines despite availability of vaccination services” [10], presents a significant challenge to successful vaccination programs globally, with developed countries inclusive [11]. Vaccine hesitancy is mainly due to various misconceptions, such as perceiving vaccines as unnecessary due to perceived self-immunity, beliefs that vaccines are not safe/effective and religious anti-vaccine beliefs due to conspiracy theories about mortality, among others [11,12,13]. Nonetheless, vaccination remains an indispensable pillar in the road to recovery from the COVID-19 pandemic, as embraced and adopted by most countries [5,14].

Slum dwellers and underserved communities are both measures of social and economic deprivation. Slum dwellers are identified as urban households lacking any of the following; adequate water and sanitation, sufficient living space, secure tenure or durable housing, similar to underserved communities, which are populations having limited or no access to resources or are otherwise deprived [15]. The COVID-19 pandemic has caused an inordinate effect among disadvantaged and underserved communities, including urban slum dwellers worldwide. The restrictive control measures enforced, particularly in the early stage of the pandemic, mostly affected the less advantaged urban poor who relied on daily income for survival [16]. In addition, slum-dwellers and underserved communities have a higher vulnerability to COVID-19 infection and morbidity compared to other advantaged or wealthier individuals [17,18]. Moreover, disparities in COVID-19 vaccination have been reported among slum-dwellers and underserved communities due to several barriers, such as long distances to vaccination centres, long queues and lack of time off work to get vaccinated, as well as low vaccine supplies [9,16]. Despite the huge efforts made to expand the COVID-19 vaccination program, successful vaccination targets still cannot be achieved without barriers and concerns faced by slum and underserved communities being addressed. This calls for a clear, in-depth assessment and understanding of COVID-19 vaccine acceptance and the unique barriers faced by this vulnerable group.

Several reviews have summarised evidence on COVID-19 vaccine acceptance and hesitancy worldwide [9,11,12,19,20] and region-wise [13,21,22], but with none focusing on slum and underserved communities. Given the increasing number of slums worldwide as a result of rapid urbanisation and other factors [23], special consideration of this population group in vaccination programs is key in preventing the uncontrolled spread of the virus and the emergence of new virus strains [16,17]. In other words, successful control of the COVID-19 pandemic warrants control of the spread in such vulnerable populations, even in developed countries, to protect society. Previous reviews have noted varying rates of COVID-19 vaccine acceptance/hesitancy across countries and regions. In addition, attitudes and perceptions about COVID-19 vaccination have been reported as one of the key determinants of vaccine acceptance since they influence people’s behaviour [21]. These were also considered in this study in the slum/underserved context.

This systematic review was, therefore, conducted to synthesise and summarise the available literature on COVID-19 vaccine acceptance/hesitancy rates, attitudes and perceptions regarding vaccination and the associated factors in slum and underserved communities. This is needed to establish a solid understanding of the levels of COVID-19 acceptance/uptake, as well as barriers and reasons for vaccine hesitancy in this group, which would help to formulate tailored strategies for addressing them. The review was guided by the following research questions:

(i) What is the prevalence of COVID-19 vaccine acceptance, hesitancy and uptake among slum and underserved communities?
(ii) What are the factors associated with COVID-19 vaccine acceptance and hesitancy among slum and underserved communities?
(iii) What are the attitudes and perceptions regarding COVID-19 vaccines among slum and underserved communities?

## 2. Materials and Methods

### 2.1. Study Design

This systematic review and meta-analysis were conducted according to a pre-registered protocol in PROSPERO (CRD42023390993) and the PRISMA guideline [24]. This systematic review considered literature concerning COVID-19 vaccination among slum and underserved communities. Literature was mainly sourced from the following platforms; PubMed, Google Scholar, Scopus and Web of Science databases.

### 2.2. Search Strategy

The study used a comprehensive search using a set of appropriate keywords and MeSH terms to identify studies reporting on COVID-19 vaccination among slum and underserved communities. For consistency and precision, similar keywords were used and searched in the article titles across all search databases. A comprehensive search of published literature was done from each of the four selected databases using the combinations of key terms and Boolean operators (Table 1). These included: “vaccine”, “vaccination”, “immunisation”, “immunisation”, “slum”, “urban poor”, “disadvantaged”, “underserved”, “slum-dwellers”, “informal settlement”, “poor housing”, “perception”, “attitude”, “acceptance”, “acceptability”, “knowledge”, “hesitancy”, “COVID-19”, “Coronavirus” and “SARS-CoV-2”.

### 2.3. Inclusion Criteria

Only population-based original observational research studies (including qualitative, quantitative and mixed-method studies) reporting on COVID-19 vaccination in slum and/or underserved communities, with no restriction to country/region location, were considered in the full review. Additionally, comparative studies were considered if they included a slum or underserved community as part of their study population. We also considered pre-prints, theses and dissertations with full text available. Only English-language articles published between 1 November 2019 to 19 January 2023 were considered.

### 2.4. Exclusion Criteria

Other grey literature, including government documents/reports, newspapers, textbooks, book chapters and protocols, were excluded. Governmental documents/reports and newspapers were excluded because they might not be written for scientific purposes. Another reason for excluding this grey literature was the lack of peer review. There are, hence, concerns about the quality and reliability of this literature. In addition, intervention studies, laboratory studies, model and framework studies, validation studies and those whose study population was not slum or underserved community were excluded. All disagreements faced in the inclusion phase of the review were discussed to reach a consensus.

### 2.5. Data Extraction

Title, abstract screening and full-text reviews were independently conducted by two authors (J.K. and S.C.) following the inclusion and exclusion criteria. After the successful screening, the following information/variables were extracted from the selected articles: first author, year of publication, study location, study design, key measurements, study population, sample size, reported acceptance or hesitancy rate, uptake rates, and other relevant findings on attitude, perception, associated factors and barriers as detailed in Table 2. The extracted data were stored in a Microsoft Excel spreadsheet for statistical analysis.

### 2.6. Quality Assessment and Data Analysis

The quality of the included articles was assessed using the Mixed-Method Appraisal Tool (MMAT) version 2018, which has a detailed description of the rating [49]. Two authors independently assessed the quality of the included studies, and in case of discrepancies, a consensus was reached, also through discussion. 

First, the characteristics of studies included in the review were summarised using frequencies and percentages. Then, the pooled vaccination acceptance, hesitancy and uptake rates were categorically obtained using random-effects models and sub-grouped according to study characteristics. Heterogeneity among studies was assessed using Q-test and $I^{2}$ test. For studies that reported only the acceptance rate, the hesitancy rate was calculated by the formula (100-acceptance rate), similar to studies that reported the hesitancy rate only where the acceptance rate was obtained by the formula (100-hesitancy rate). 

Meta-regression analyses and subgroup analyses were conducted to determine whether study characteristics could explain variability across studies. This included study year (2020, 2021, and 2023, as this was related to vaccine availability), region (Africa, Americas and Asia; related to vaccination policies adopted by different countries), sample size (<1000 and >1000) and study population (general and non-general-this included parents, hospital patients and healthcare workers). Only study variables with meaningful and practical categories were considered. We assessed whether vaccination acceptance, hesitance and uptake varied according to the selected study variables by univariate meta-regression. Significant variables (*p* < 0.05) were then included in the multivariable meta-regression model. 

In addition, sensitivity analysis was done by considering only studies with good methodological quality and studies published before and after the median publication year. The presence of publication bias was visualised by funnel plots to measure the asymmetry and quantitatively examined with Egger’s linear regression test. We used the trim-and-fill method to adjust for potential publication bias. The meta-analyses were done using the meta-prop package (method = Inverse and summary measure = PLOGIT) of R Studio (version 4.2.1). In addition, other key findings on attitudes and perceptions, as well as associated factors and reasons for vaccine hesitancy, were assessed and summarised thematically.

## 3. Results

### 3.1. Study Selection and Search Results

**Figure 1:**
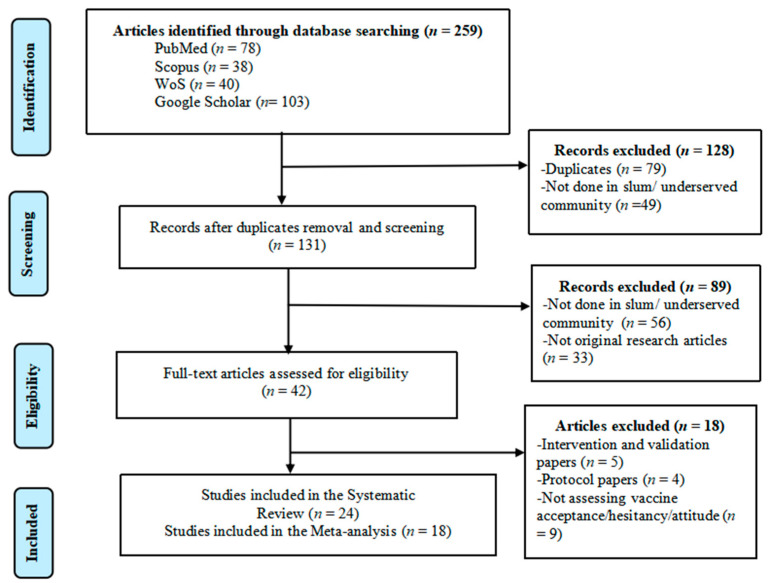
PRISMA flow diagram showing search strategy and study selection process.

The PRISMA diagram (Figure 1) illustrates the selection process and shows the reasons for exclusion. A total of 259 articles were identified from the initial search, and 131 remained after removing duplicates and title screening. On further screening, 42 articles remained for eligibility assessment after excluding those which were not original studies and not done in slum/underserved communities. The final assessment yielded 24 articles for further analysis [25,26,27,28,29,30,31,32,33,34,35,36,37,38,39,40,41,42,43,44,45,46,47,48].

### 3.2. Characteristics of the Included Studies

The twenty-four (24) included studies were published between 2021–2023, with nine articles published in 2021, 14 in 2022 and one in 2023. Eleven (11) studies were conducted in Asia, nine in North America, three in Africa, and one in South America. The studies represent seven countries, with nine from the USA, five from Bangladesh and four from India. Other countries included Pakistan (2 studies), Uganda (2), Brazil (1), and Kenya (1).

Nineteen (19) of the studies were quantitative, three were qualitative, and two were mixed methods. The studies included in this review comprised 30,323 participants with a sample size ranging from 34 to 12,887 (mean = 1263.5, SD = 2610.3). Regarding the study population, the majority (21 studies) were from the general slum/ underserved population, and the rest were from hospital patients, healthcare workers and parents (one study each) (Table 3). 

The overall quality of the studies was generally good, implying that the included studies satisfied most of the quality criteria. However, lower scores in item 4 (non-response bias-45.5%) were noted among most quantitative studies, as detailed in Supplementary Table S1A,B.

### 3.3. Primary Findings

**Figure 2:**
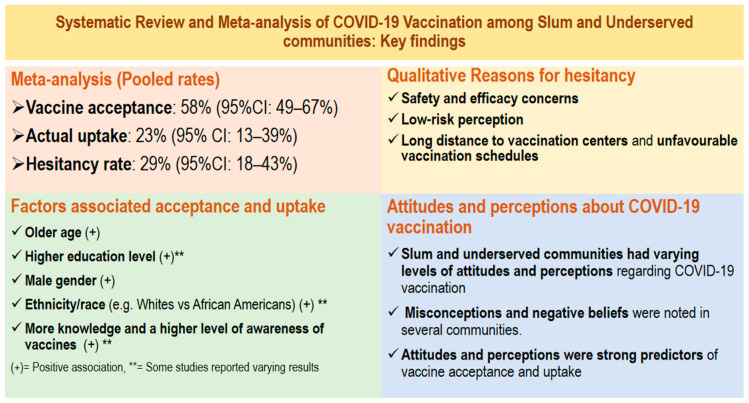
Main findings from the Systematic Review and meta-analysis of COVID-19 vaccination among slum and underserved communities.

**Figure 3:**
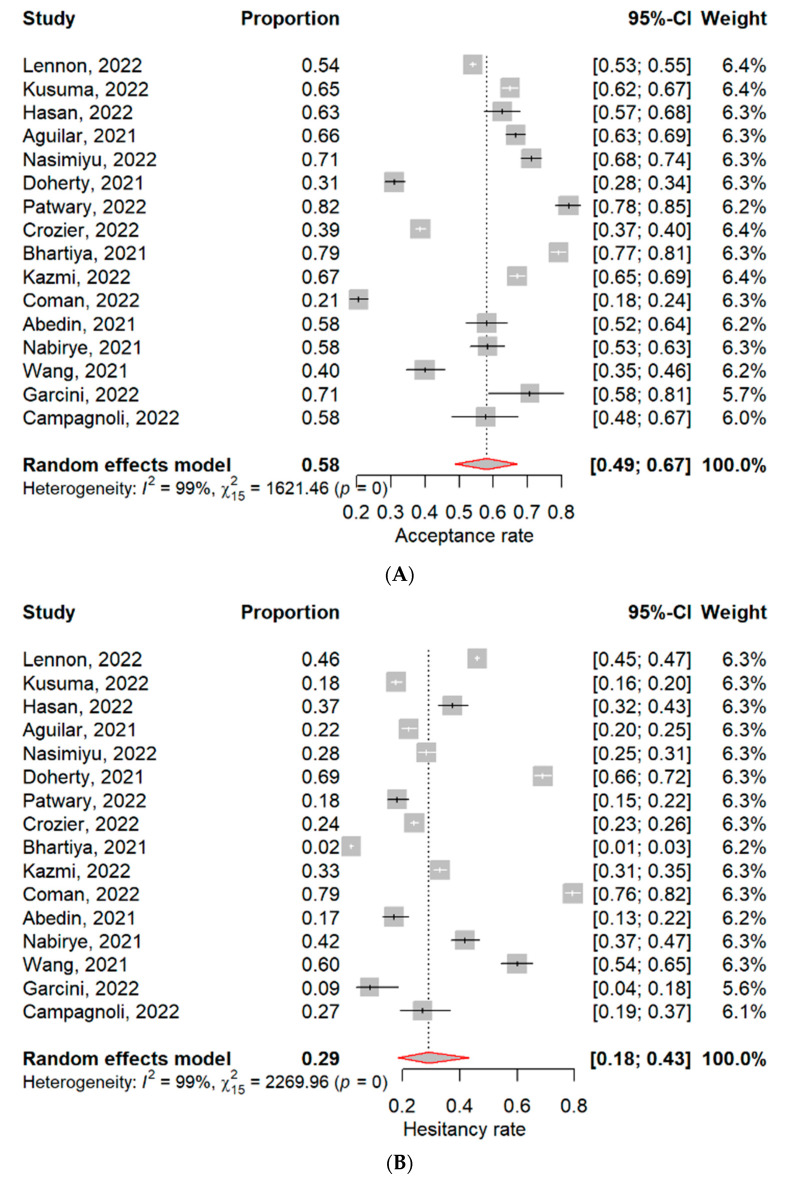

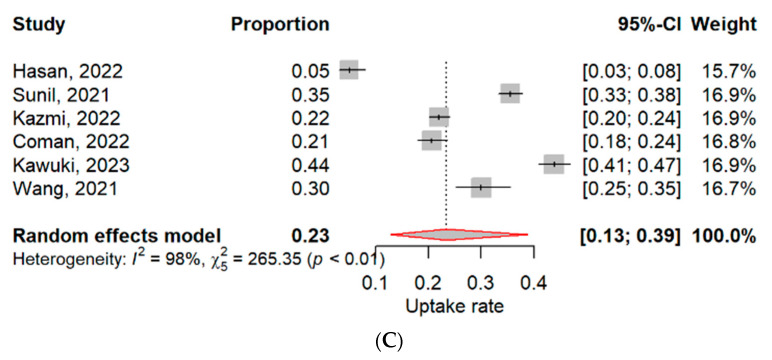
Forest plots for pooled (**A**) Acceptance rate, (**B**) Hesitancy rate and (**C**) Uptake rate of COVID-19 vaccine among slum and underserved communities [25,26,27,28,29,30,31,32,33,34,35,36,37,38,39,40,41,42,43,44,45,46,47,48].

The overall findings of this systematic review and meta-analysis are summarised in Figure 2. Detailed findings of vaccine acceptance, hesitancy and uptake are in Figure 3, associated factors and reasons for vaccine hesitancy in Table 4, and attitudes and perceptions are detailed in Table 2.

#### 3.3.1. COVID-19 Vaccine Acceptance and Hesitancy 

Sixteen (16) studies reported on COVID-19 vaccine acceptance and hesitancy (Figure 3A,B). The overall acceptance was 58% (95% CI: 49–67%), ranging from 21% to 82.0%, and with very high significant heterogeneity (*I*^2^ = 99%, *p* < 0.01), Figure 3A. The overall vaccine hesitancy was 29% (95% CI: 18–43%), ranging from 2% to 79%, also with very high heterogeneity (*I*^2^ = 99%, *p* < 0.01), Figure 3B. The highest vaccine hesitancy was reported in the USA, with a pooled prevalence of 45%, followed by Uganda (42%) and Pakistan (33%). However, vaccine acceptance increased from 2021 (56%) to 2022 (59%), opposite to vaccine hesitancy which declined from 35% to 32% in the same period (2021–2022). Vaccine acceptance was highest among healthcare workers (71%), followed by parents (67%) and hospital patients (58%).

Meta-regression and subgroup analyses were done to see whether study year, region, study population, and sample size could explain the observed heterogeneity among acceptance and hesitancy studies. Univariate meta-regression showed that vaccine acceptance was positively associated with region and study population (*p* = 0.01 and <0.01), but vaccine hesitancy had a negative association with the two variables (both *p* = 0.01). After multivariate meta-regression, only vaccine acceptance was positively associated with the study population (*p* = 0.03) (Table 4).

In subgroup analyses, only the study population explained some of the variability among acceptance studies, with no significant heterogeneity among non-general population studies (three studies). Moreover, Asia and Africa had higher vaccine acceptance rates than the Americas (70% and 65% vs 47%), similar to non-general population studies compared to general population studies (65% vs 56%). Contrariwise, the Americas had higher vaccine hesitancy than Africa and Asia (40% vs 35% and 17%), similar to general population studies compared to non-general population studies (32% vs 19%) (Table 4). 

Sensitivity analyses by considering only studies done before and after the median year (six studies) showed lower acceptance (56%, 95% CI: 41–70%) and hesitancy (27%, 95% CI: 9–59%) rates, both with high heterogeneity (both *I*^2^ = 99%, *p* < 0.01). In addition, considering only studies with good quality (14 studies, after removing two studies [38,40]) gave higher acceptance (59%, 95% CI: 51–67%) and hesitancy (31%, 95% CI: 22–40%) rates, both with significant heterogeneity (both *I*^2^ = 99%, *p* < 0.01).

**Figure 4:**
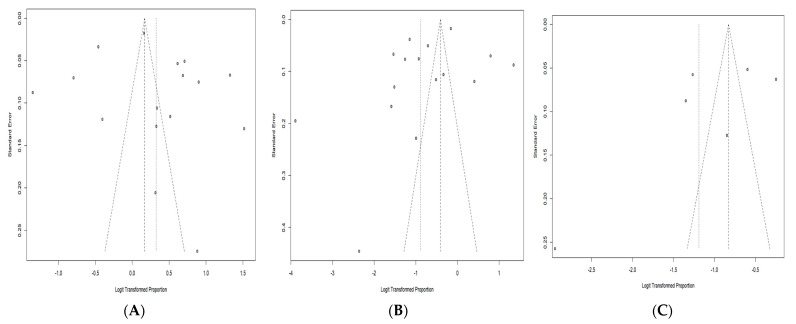
Funnel plots of (**A**) Acceptance studies (Egger’s test, *p* = 0.504), (**B**) Hesitancy studies (Egger’s test, *p* = 0.209) and (**C**) Uptake studies (Egger’s test, *p* = 0.291).

Publication bias was assessed in the acceptance and hesitancy articles using visual inspection of the funnel plot, which showed a slight asymmetry in the studies, implying probable publication bias toward studies with low acceptance rates and high hesitancy rates (Figure 4A,B). However, further evaluation using Egger’s test showed no significant publication bias in acceptance and hesitancy studies (*p* = 0.504 and 0.209). Nevertheless, when the trim-and-fill analyses were executed, the adjusted acceptance and hesitancy rates were 52.6% (95% CI: 42.8–62.2 %) and 42.1% (95% CI: 27.1–58.7) after filling in three and five missing studies, respectively.

#### 3.3.2. COVID-19 Vaccine Uptake 

Six (6) studies reported on the actual uptake/receipt of the COVID-19 vaccine among slum and underserved communities (Figure 3C). The overall uptake was 23% (95% CI: 13–39%), ranging from 5% to 44%, and with very high significant heterogeneity (*I*^2^ = 98%, *p* < 0.01). Uganda reported the highest vaccine uptake (44%), followed by India (35%) and the USA (25%). Vaccine uptake was lowest in Bangladesh (5%). Uptake rates increased from 2021 (33%) to 2023 (44%). 

Univariate meta-regression revealed that vaccine uptake was positively associated with the study year (*p* < 0.01) but negatively associated with the region (*p* < 0.01). On multivariate meta-regression, vaccine uptake was only significantly associated with study year but with a negative association (*p* = 0.04), Table 4.

Subgroup analyses showed that only the study year explained some of the variability among vaccine uptake studies, with no significant heterogeneity among 2021 studies (2 studies). Additionally, 2021 studies reported higher vaccine uptake compared to 2022 studies (33% vs 14%), similar to studies from the Americas compared to Asian studies (25% vs 17%), Table 4.

Sensitivity analyses by considering only vaccine uptake studies done before and after the median year (three studies) showed a higher uptake rate of 37% (95% CI: 29–45%), with significant heterogeneity (*I*^2^ = 93%, *p* < 0.01). However, considering only studies with good quality (four studies, after removing two studies [29,40]) gave a slightly lower uptake rate of 21% (95% CI: 8–45%), also with significantly high heterogeneity (*I*^2^ = 99%, *p* < 0.01).

Publication bias among vaccine uptake articles was also assessed using visual inspection of the funnel plot, which showed a slight asymmetry in the studies, implying probable publication bias toward studies with higher uptake rates (Figure 4C). Further evaluation by Egger’s test found no significant publication bias among uptake studies (*p* = 0.291). Nevertheless, when the trim-and-fill analysis was executed, the adjusted uptake rate was 30.1% (95% CI: 14.4–52.3%) after filling in one missing study.

#### 3.3.3. Factors Associated with COVID-19 Vaccination

Seventeen (17) studies reported on the factors associated with COVID-19 vaccination (acceptance/hesitancy and uptake), Table 5. Older age was a strong predictor reported in nine studies, with some reporting a positive association with acceptance and uptake [28,30,35,38,41,42,47] and a negative association in others [27,29]. High education level and good health literacy were also positive correlates of vaccine acceptance and uptake [25,29,37,39,42], though some studies reported a negative association [32,38]. Male gender had a positive association with vaccine acceptance in five studies [25,29,33,35,38]. Ethnicity/race was a strong predictor in the USA and reported in five studies, with more vaccine hesitancy among ethnic minorities, mostly among Blacks and Hispanics [25,33,35,45,47]. Ethnicity/tribe was also a strong predictor in Uganda [41]. Being employed and having higher SES had a positive association with acceptance and uptake [29,39], while occupation had a varying effect on vaccination [29,37,38,42]. Good knowledge and awareness of the vaccines as well as reliable information sources, were strong positive associates of vaccine acceptance and uptake [28,34,37,41]. Perceived benefit of vaccination, high perceived susceptibility and severity of COVID-19, and self-efficacy to receive vaccination were also strong positive predictors of vaccine acceptance and uptake [27,30,34,39,41,48], same as cues to action in the forms of text messages and reminders for people to get vaccinated [41,48]. However, perceived barriers such as serious side effects and safety concerns of the vaccines and long distances to vaccination centres were associated with higher vaccine hesitancy, same as negative attitudes, beliefs and perceptions about the vaccine, for example, anti-vaccine attitudes and religious beliefs against vaccination [28,33,34,37,39,41,48]. Trust and confidence in the vaccine, healthcare workers and the government were also reported in several studies as strong positive predictors [33,34,42,47,48]. Other reported factors with a positive association included urban residence [25,35], COVID-19 infection and test history, and vaccination of other family members [39,45]. Having chronic diseases [28,42] and being religious [29,41] showed varying effects on vaccination.

#### 3.3.4. Qualitative Reasons for COVID-19 Vaccine Hesitancy

Nine (9) studies reported on possible reasons for COVID-19 vaccine hesitancy among slum and underserved communities (Table 5). Concerns about vaccine safety, possible side effects and efficacy/effectiveness of vaccines were the most reported reasons (eight studies) for not getting vaccinated [29,30,31,32,33,36,46,47]. The low-risk perception was also a common reason reported in three studies [27,30,32], same as negative beliefs, attitudes and misconceptions about COVID-19 vaccines [27,32,46]. In addition, long distances to vaccination centres, inability to spare a day from work and missed opportunity [36,47], limited information on vaccination [32,46], and mistrust of healthcare workers, government and manufacturers [33,46,47] were also reported reasons for hesitancy.

#### 3.3.5. Attitudes and Perceptions about COVID-19 Vaccination

Eleven (11) studies reported on attitudes and perceptions regarding COVID-19 vaccination (Table 2). Varying levels of knowledge and awareness of COVID-19 vaccination have been reported among the included studies. Higher awareness of COVID-19 vaccination (over 60%) was reported in Kenya but with rates higher in Asembo than in Kibera slums [32]. However, lower rates of knowledge and awareness (less than 10%) were reported in India (Mumbai) [38] and Uganda [43]. Knowledge and awareness of vaccination were associated with age, gender, marital status, education, income, occupation, and socioeconomic status [26,37,38,43].

Regarding attitudes and perceptions, COVID-19 vaccines were perceived as safe, effective and important by the majority in underserved communities of the USA [31], similar to Ugandan and Bangladeshi slum communities [43,45]. However, a lack of trust in vaccines and doubt of vaccines’ safety and effectiveness were reported in India and Bangladesh [36,44]. In Ugandan slum communities, the fear of being unable to access services in the future due to being unvaccinated was the dominant motivation for vaccination [43], while in Bangladesh, acceptance of the COVID-19 vaccine among slum residents improved with time after seeing more and more community members getting vaccinated and knowing that vaccination was free of cost [26]. Slum and underserved communities had the lowest acceptance and uptake rates compared to other residences [42] due to structural inequities in the vaccination that affected access to COVID-19 vaccination, and, thus, the majority preferred decentralised local vaccine camps to receive vaccines rather than going to central vaccination centres [26,28,46]. In the USA, residents of underserved communities believe that ensuring good knowledge and access to reliable information, providing bilingual staff to administer the vaccine, and giving an option for mobile/home vaccination would facilitate vaccine uptake [46]. 

Attitude towards COVID-19 vaccination was associated with religious beliefs and cultural norms, ethnicity/race, education level, income, cues from social ties, and clinical, trust, and religious/spiritual barriers [37,40], while perception was associated with age, gender, education, marital status, and family size [44].

## 4. Discussion

### 4.1. Primary Findings 

To our knowledge, this is the first systematic review to summarise evidence on COVID-19 vaccination among slum and underserved communities. The review first analysed the vaccine acceptance, hesitancy and uptake rates. Then it assessed factors associated with vaccination and reasons for hesitancy. It additionally assessed attitudes and perceptions of COVID-19 vaccination among this vulnerable population. All these are strong contributions to literature. Moreover, in addition to the multi-dimensional and comprehensive approach, we used a reproducible search with a well-established keyword system for the identification of studies from key databases, all of which were strengths of this systematic review.

The review found that vaccine acceptance was 58% (95% CI: 49–66%), hesitancy 29% (95% CI: 18–43%) and uptake 23% (95% CI: 13–39%). Vaccine acceptance and uptake were associated with various sociodemographics, including age, education level, gender, and ethnicity, among others. Moreover, safety and efficacy concerns, low-risk perception and long distance to vaccination centres were the main reasons for hesitancy. Results also indicate varying levels of attitudes and perceptions regarding COVID-19 vaccination, with misconceptions and negative attitudes reported among slum and underserved communities. With these findings, the review comprehensively addressed the research questions.

The review showed that although 58% of residents of slum and underserved communities were willing to take up the COVID-19 vaccine, only 23% had actually received the vaccine. The observed uptake rate in this study is much lower than the current global vaccination rate of 69% [5], which is probably due to the various structural barriers to vaccine access in slums and underserved communities [9]. Moreover, although results indicated an increase in acceptance rates since the vaccine rollout, hesitancy in these less advantaged communities (29%) is still substantially high, although similar to previous studies elsewhere [21]. Notably, the highest vaccine hesitancy was reported in underserved communities of the USA (45%), which is similar and consistent with findings of previous reviews [9,11,21]. 

The review results also indicate that COVID-19 vaccine acceptance, hesitancy, and uptake significantly varied according to region, study population, and study year. The region had a varying effect on vaccine acceptance, hesitancy and uptake, with more acceptance rates reported in Asia and Africa compared to the Americas. However, the Americas had higher vaccine uptake compared to Asia. The findings may be explained by the different vaccination policies in various countries, for example, mandatory vaccination, travel restrictions and lockdowns [3,4]. These various policies could indirectly affect people’s perceptions and attitudes toward vaccines in that particular region or country [12,20], thus, the observed trend among the analysed studies.

The study population also had a varying effect on vaccine acceptance and hesitancy, with studies done among parents, hospital patients and healthcare workers reporting more vaccine acceptance compared to general population studies. Various population groups tend to have varying perceived susceptibility to COVID-19, which could affect their acceptance and hesitancy of the COVID-19 vaccine [10,13,16], thus, the observed trend. 

Furthermore, study year positively affected vaccine uptake, with most recent studies (done in 2023) reporting higher uptake rates compared to early studies. Vaccine availability, which was introduced around early 2021 and became more accessible in later stages of the pandemic [5,8], might explain the observed trend. Moreover, the varying COVID-19 situation (i.e., changes in new confirmed cases and deaths over time) and the prevalent virus subtype (such as Delta and Omicron, among others), which present with varying infectivity and severity [1] may also influence people’s willingness to get vaccinated. 

The review highlighted several barriers which might explain the observed low uptake and high hesitancy rates. The most common reasons for vaccine hesitancy among these communities include safety and efficacy concerns, low-risk perception, long distance to vaccination centres and unfavourable vaccination schedules. Such barriers have also been reported in other non-slum communities [11,13,22] and, thus, should be considered and addressed with a customised approach for successful vaccination programs. Infodemic management, risk communication and continued vaccine education to clear safety concerns are, thus, warranted. Moreover, considering flexible vaccination options such as mobile vaccination units where people can be vaccinated at their homes or workplace would solve access barriers faced among slum and underserved communities [46,50].

The study highlighted several factors associated with COVID-19 vaccination among the study population. These included personal (e.g., age, gender, race/ethnicity, education, attitude and perceptions), interpersonal (e.g., employment, occupation, SES, household size, cues to action) and structural (e.g., knowledge, information sources, distance to vaccination centres, beliefs, religion) factors. These determinants have also been reported among non-slum communities in previous reviews [12,13,20,21,22]. The most common determinants in our study were age, education level, gender, ethnicity/race, and knowledge and awareness of vaccines, among others. For successful vaccination programs in these communities, such sociodemographic factors should be considered and incorporated when designing targeted interventions. Current and future vaccine education initiatives should, thus, target young, less-educated women and ethnic minorities.

Study results also indicate that residents of slum and underserved communities had varying levels of attitudes and perceptions regarding COVID-19 vaccination, with misconceptions and negative beliefs noted in several communities. Notably, attitudes and perceptions were strong predictors of vaccine acceptance and uptake in this study. To reduce the hesitancy rates, vaccine education programs should, thus, be tailored to address existing misconceptions and negative beliefs about COVID-19 vaccines while leveraging positive attitudes and perceptions.

### 4.2. Limitations and Future Research

This systematic review has some limitations. The estimated vaccine acceptance, hesitancy, and uptake rates could have been affected by the persistent heterogeneity. Although we performed subgroup analysis and sensitivity analysis, the observed heterogeneity could not be fully addressed/explained. However, it should be noted that these studies were from different countries with varying COVID-19 situations and vaccination policies and used different data collection methods, making heterogeneity unavoidable. The small number of uptake studies could have affected the true estimation of the effect size and meta-regression results. Although we used a comprehensive keyword search strategy, some relevant studies might still have been missed out since only articles in the English language were considered. Additionally, we only considered studies from slum and underserved communities, and studies that used rural population without specifying that it is underserved were not considered. Future studies are needed to summarise evidence on COVID-19 vaccination, specifically in rural areas. Due to the self-report nature of the studies used in this review, there is a risk of recall and social-desirability bias. No studies were identified from developed countries apart from the USA, although we used a systematic search using PRISMA guidelines. Future studies should, thus, focus on other developed countries to explore vaccination uptake among their underserved and slum communities. Despite the limitations, the study provides valuable insights into COVID-19 vaccination, associated factors, and barriers among slum and underserved communities.

## 5. Conclusions

This systematic review, to our knowledge, provides the first summarised evidence on COVID-19 vaccination, including attitudes and perceptions, associated factors, and barriers among slum and underserved communities. The findings indicate that although more than half of residents in slum and underserved communities were willing to take the vaccine, only a third had been vaccinated. Therefore, there is a need for continuous vaccine education and infodemic management to address existing misconceptions and negative beliefs. Such education programs should focus on the young, less educated, women, and ethnic minorities. Addressing access barriers, for example, by availing mobile vaccination units to vaccinate people at home or workplaces, would increase vaccine uptake among those willing to be vaccinated. 

## Figures and Tables

**Table 1:** Key terms or Boolean operators used for search.

Search	Search Terms (Boolean Operators)
1	“COVID-19 vaccine” OR “COVID-19 vaccination” OR “COVID-19 immunisation” OR “COVID-19 immunisation AND “slum” OR “urban poor” OR “disadvantaged” OR “underserved” OR “slum-dwellers” OR “informal settlement” OR “poor housing”
2	“SARS-CoV-2 vaccine” OR “SARS-CoV-2 vaccination” OR “SARS-CoV-2 immunisation” OR “SARS-CoV-2 immunisation AND “slum” OR “urban poor” OR “disadvantaged” OR “underserved” OR “slum-dwellers” OR “informal settlement” OR “poor housing”
3	“Coronavirus vaccine” OR “Coronavirus vaccination” OR “Coronavirus immunisation” OR “Coronavirus immunisation AND “slum” OR “urban poor” OR “disadvantaged” OR “underserved” OR “slum-dwellers” OR “informal settlement” OR “poor housing”
4	“COVID-19 vaccine acceptance” OR “COVID-19 vaccine hesitancy” OR “COVID-19 vaccine acceptability” AND “slum” OR “urban poor” OR “disadvantaged” OR “underserved” OR “slum-dwellers” OR “informal settlement” OR “poor housing”
5	“COVID-19 vaccine attitude” OR “COVID-19 vaccine knowledge” OR “COVID-19 vaccine perception” AND “slum” OR “urban poor” OR “disadvantaged” OR “underserved” OR “slum-dwellers” OR “slum dwellers” OR “informal settlement” OR “poor housing”

COVID-19 = Coronavirus Disease 2019.

**Table 2:** Characteristics of included studies.

Author, Year	Study Setting /Country	Study Type and Key Measures	Study Population	Sample Size	Vaccine Acceptance/Hesitancy/Uptake Rates	Relevant Findings
Lennon et al., 2022 [25]	USA	Cross-sectional -Acceptance of COVID-19 booster, influenza and combination influenza-COVID-19 booster vaccines	Underserved communities	12,887	Acceptance: 45% for a COVID-19 booster alone; 58% for an influenza vaccine alone; 50% for a combination vaccine.	There was a lower acceptance among female, Black/African American, Native American/American Indian and rural respondents. Higher acceptance was noted among those with college and post-graduate degrees.
Alam et al., 2022 [26]	Bangladesh(Dhaka)	-Qualitative study -Perceptions and attitudes	Urban slum dwellers	36	N/A	Acceptance of COVID-19 vaccines improved with time after seeing more and more community members getting vaccinated and knowing that vaccination was free of cost Women knew more about COVID-19 vaccination compared with men, same as youths (aged 18–24 years) compared with older age groups. Structural inequities in the vaccination program, e.g., complicated online registration system and long queues at vaccination centres, meant that many urban poor could not access COVID-19 vaccination as they worried about missing a day’s work
Kusuma et al., 2022 [27]	India (Delhi)	-Cross-sectional -Acceptance and determinants	Urban slum communities	1539	Acceptance: 64.9%; hesitancy: 17.7%; not sure: 17.4%	Reasons for hesitancy were: the belief that they had immunity, COVID-19 was a hoax, the vaccine was not necessary and they did not want to disturb the natural bodily systems by the vaccine. Older age, low perceived susceptibility and severity of COVID-19, low self-efficacy to protect against COVID-19 and unawareness and non-use of the Aarogya Setu App were significant predictors of vaccine hesitancy
Hasan et al., 2022 [28]	Bangladesh (Dhaka)	-Cross-sectional -Acceptance and determinants	Urban slum dwellers	318	-Uptake: 5%; acceptance: 62.6%	A majority (58%) preferred local vaccine camps to receive vaccines rather than going to the designated healthcare facilities. Older age, having adequate knowledge regarding COVID-19 symptoms, comorbid patients in the households, no religious misconceptions and no doubt about the safety of the vaccine was associated with higher odds of vaccine acceptance
Sunil et al., 2021 [29]	India (Bengaluru)	Cross-sectional -Uptake and associated factors	Urban slum dwellers	1638	-Uptake: 35.5%	Occurrence of mild or serious adverse effects was the dominant factor for vaccine hesitancy and was more reported among women than men across all age groups. Vaccine uptake was high among the youth (18–45 years), males, Christians, graduates, clerical and skilled workers and the upper middle socioeconomic class.
Aguilar et al. 2021 [30]	Brazil (Salvador)	Cross-sectional -Acceptance and determinants	Urban slum dwellers	985	Acceptance: 66.0%; hesitancy: 26.1%; not sure: 7.9% -Parental acceptance for children: 67%; hesitancy: 18%; 15% unsure	The main reasons for hesitancy were concerns about vaccine efficacy and potential side effects. The main reasons for acceptance were the high incidence of COVID-19 cases and perceived susceptibility. COVID-19 vaccine hesitancy was associated with younger age and low social capital, i.e., low perceived importance of vaccination to protect one’s family, friends and community. Parental acceptance of their children’s vaccination was positively associated with acceptance among parents themselves.
Cohrs et al., 2022 [31]	USA (Ohio)	Cross-sectional, hospital-based -Barriers and experience	Underserved patients	189	N/A	A majority (77%) agreed that COVID-19 vaccines were safe, effective and important for the health of others in their community. Adverse effects and the cost of the COVID-19 vaccine were noted to be significantly more of a concern with the COVID-19 vaccine than the influenza vaccine
Nasimiyu et al., 2022 [32]	Kenya (Kibera and Asembo)	Cross-sectional -Acceptance and determinants	Urban slum dwellers	856	Acceptance: 83.6% in Asembo and 59.8% in Kibera	Awareness of the COVID-19 vaccine existence was higher in Asembo (89.7%) than in Kibera (66.8%) Reasons for vaccine hesitancy were safety concerns, insufficient information available to decide, low-risk perception and a lack of belief in the vaccine. Post-secondary education was associated with fewer odds of vaccine acceptance compared to those without education
Doherty et al., 2021 [33]	USA (North Carolina)	Cross-sectional -Hesitancy and correlates	Underserved communities	948	Overall hesitancy was 68.9%—Whites: 62.7%; Blacks: 74%; Latinx: 59.5%	The common reason for hesitancy was safety and efficacy concerns and gov’t mistrust Significantly more Blacks (28.6%) mistrusted the government compared to Whites (17.9%) and Hispanics (13.3%). Being female, being Black, calendar month, safety concerns and government distrust were associated with higher odds of vaccine hesitancy
Patwary et al., 2022 [34]	Bangladesh (Dhaka and Khulna)	Cross-sectional -Acceptance determinants (antecedents)	Urban slum dwellers	400	Acceptance: 82%	Most of the slum dwellers who were confident, complacent, calculative, and responsible showed a higher vaccine acceptance rate. Those who had no anti-vaccine attitudes and obtained vaccine information from the newspaper were highly willing to accept the COVID-19 vaccine. Gender, marital status, education level, occupation status, monthly family income, long-standing illness, perceived health condition and smoking behaviour were significantly associated with vaccination antecedents
Crozier et al., 2022 [35]	USA (Alabama)	Cross-sectional -Acceptance and correlates	Underserved communities	3721	Acceptance: 38.7%; hesitancy: 24.1%; not sure: 37.2%	Sex (male), race/ethnicity, older age and residence (rural/urban) were associated with vaccine acceptance
Tamysetty et al., 2021 [36]	India (Mumbai, Bengaluru, Kolkata and Delhi)	Cross-sectional Mixed methods -Facilitators and barriers to vaccination	Urban slum dwellers	296	N/A	Less than half (44.6%) had trust in COVID-19 vaccines The main reasons for not getting vaccinated were fear of possible side effects, the uncertainty of getting the vaccine, safety concerns of vaccines, long distance to vaccination centre and inability to spare a day from work, among others
Qasim et al., 2022 [37]	Pakistan (Karachi)	Cross-sectional -Qualitative study -Perceptions and experience of vaccination	Urban slum dwellers	46	N/A	Religious beliefs and cultural norms influenced attitudes toward COVID-19 and vaccination Awareness about the COVID-19 vaccine was influenced by sex, educational status and socioeconomic status. Vaccine hesitancy was linked to personal belief systems, vaccine mistrust and public perceptions Vaccine acceptance was linked to knowledge and awareness about the vaccine and trusted sources of information Participants with good health literacy and those from healthcare backgrounds were more likely to share views that indicated vaccine acceptance.
Bhartiya et al., 2021 [38]	India (Mumbai)	Cross-sectional -Knowledge, attitude and perception	Urban slum dwellers	1342	Acceptance: 79%; hesitancy: 2%; unsure: 19%	A majority (91%) were unaware of vaccine availability, and awareness was associated with age, gender, education, income and occupation Acceptance was associated with age, gender, education, and occupation
Kazmi et al., 2022 [39]	Pakistan (Islamabad and Rawalpindi)	Cross-sectional -Uptake, acceptance and determinants	Urban slum dwellers	1760	Uptake: 16% partially; 6% fully vaccinated; acceptance: 67%	Uptake and acceptance were associated with higher education, being employed, prior infection in the family (but not self), family vaccination, knowing of and living close to a vaccination centre and being worried about COVID-19.
Coman et al., 2022 [40]	USA	Cross-sectional -Attitude and perception	Underserved communities	795	Uptake: 20.6%; hesitancy: 79.4%	Ethnicity/ race (Black), less education level, Clinical, trust, and religious/spiritual barriers were negatively related to attitudes toward vaccination. Cues from authority and social ties, and high income were positively associated with vaccination attitudes.
Kawuki et al., 2023 [41]	Uganda	Cross-sectional -Comparative study -Uptake and determinants	Urban slum dwellers and estate residents	1025	Uptake: 43.8% fully vaccinated in slums compared to 39.9% in estate	Uptake was positively associated with knowledge level, perceived benefits and cues to action. Perceived barriers such as serious side effects and long distances, and depressive symptoms were negatively associated with uptake
Abedin et al., 2021 [42]	Bangladesh	Cross-sectional -Acceptance and correlates	Urban slum dwellers	253	Acceptance: 58.1%; hesitancy 17%; not sure: 24.9%	Slum residents had the lowest acceptance rate compared to other residences (68.7–81.5%) Hesitancy was associated with older age, low education, being a day labourer, having chronic diseases and low confidence in the country’s healthcare system
Nabirye et al. 2021 [43]	Uganda	Cross-sectional -Acceptance, knowledge and perceptions	Urban slum dwellers	367	Acceptance: 58.3%	90.5% had insufficient knowledge about COVID-19 vaccines. About 50% thought that the vaccine was safe and that everyone should get vaccinated. Fear of being unable to access services in the future because of not being vaccinated was the most common motivation for vaccination. Marital status, age and education level were significantly associated with knowledge.
Mamun et al., 2021 [44]	Bangladesh (Dhaka)	Cross-sectional -Perceptions	Urban slum dwellers	434	N/A	The majority attest to the importance of getting vaccinated but had a doubt regarding vaccine effectiveness and safety and, thus, were not quite confident about taking the vaccine. Perception varied with family size, education, gender, marital status, and age.
Wang et al., 2021 [45]	USA (Delaware)	Cross-sectional -Uptake and determinants	Underserved communities	293	Uptake: 30%; hesitancy: 60%	Being black was associated with vaccine hesitancy COVID test history was linked to low vaccine hesitancy.
Garcini et al., 2022 [46]	USA (South Texas)	Cross-sectional -Mixed methods -Barriers and facilitators	Community Health Workers in Underserved Communities	64	Acceptance: 70.7%; Hesitancy: 8.6%; unsure: 20.7%	Barriers to vaccination included mistrust of manufacturers and administrators, concerns about vaccine safety, fear of discrimination/stigmatisation from HCWs administering the vaccine, fear of exploitation/manipulation by the government or health authorities, and having personal information mishandled. Additional barriers included being undocumented, fear-inducing myths and beliefs, limited information and logistics about vaccination access Ensuring good knowledge and accessible information about COVID-19 vaccines, bilingual staff to administer the vaccine, convenient locations and vaccination schedules, short wait times, giving the option to get vaccinated at home and confidentiality were key facilitators.
Campagnoli et al., 2022 [47]	USA (Chicago)	Cross-sectional, Hospital-based -Acceptance and drivers	Underserved Patients	97	Acceptance: 57.8%; hesitancy: 27%	Acceptance varied with age, race, household size, and trust in healthcare workers Among those willing to receive the vaccine, the main driver of being unvaccinated was missed opportunity (not having access to a doctor or not seen a doctor lately) Reasons for hesitancy included: fear of short- and long-term side effects, not enough research on the COVID-19 vaccines, the belief that it is not effective, and government mistrust
Robinson et al., 2022 [48]	USA (Alaska and Idaho)	Cross-sectional, Qualitative study -Factors for hesitancy	Underserved communities	34	N/A	Vaccine uptake was influenced by vaccine confidence, complacency (perceived susceptibility, severity, and benefits) and convenience (access, availability and messaging)

N/A = Not available, COVID-19 = Coronavirus Disease 2019, USA = United States of America.

**Table 3:** Summary of COVID-19 vaccine acceptance, hesitancy and uptake from the included studies.

Author	Year	Country	Study Design	Population	Sample Size	Acceptance (%)	Hesitancy (%)	Uptake (%)
Lennon et al. [25]	2022	USA	Quantitative	General	12,887	54	46	--
Alam et al. [26]	2022	Bangladesh	Qualitative	General	36	--	--	--
Kusuma et al. [27]	2022	India	Quantitative	General	1539	64.9	17.7	--
Hasan et al. [28]	2022	Bangladesh	Quantitative	General	318	62.6	37.4	5
Sunil et al. [29]	2021	India	Quantitative	General	1638	--	--	35.5
Aguilar et al. [30]	2021	Brazil	Quantitative	Parents	985	66.5	22.1	--
Cohrs et al. [31]	2022	USA	Quantitative	General	189	--	--	--
Nasimiyu et al. [32]	2022	Kenya	Quantitative	General	856	71.1	28.3	--
Doherty et al. [33]	2021	USA	Quantitative	General	948	31.1	68.9	--
Patwary et al. [34]	2022	Bangladesh	Quantitative	General	400	82	18	--
Crozier et al. [35]	2022	USA	Quantitative	General	3721	38.7	24.1	--
Tamysetty et al. [36]	2021	India	Mixed-method	General	296	--	--	--
Qasim et al. [37]	2022	Pakistan	Qualitative	General	46	--	--	--
Bhartiya et al. [38]	2021	India	Quantitative	General	1342	79	2	--
Kazmi et al. [39]	2022	Pakistan	Quantitative	General	1760	67	33	22
Coman et al. [40]	2022	USA	Quantitative	General	795	20.6	79.4	20.6
Kawuki et al. [41]	2023	Uganda	Quantitative	General	1025	--	--	43.8
Abedin et al. [42]	2021	Bangladesh	Quantitative	General	253	58.1	17	--
Nabirye et al. [43]	2021	Uganda	Quantitative	General	367	58.3	41.7	--
Mamun et al. [44]	2021	Bangladesh	Quantitative	General	434	--	--	--
Wang et al. [45]	2021	USA	Quantitative	General	293	40	60	30
Garcini et al. [46]	2022	USA	Mixed-method	Healthcare workers	64	70.7	8.6	--
Campagnoli et al. [47]	2022	USA	Cross-sectional	Hospital patients	97	57.8	27	--
Robinson et al. [48]	2022	USA	Qualitative	General	34	--	--	--
	2021: 9 2022: 14 2023: 1	USA: 9 Bangladesh: 5 India: 4 Brazil: 2 Uganda: 2 Pakistan: 1 Kenya: 1	Quantitative: 19 Qualitative: 3 Mixed methods: 2	General: 21 Healthcare workers: 1 Hospital patients: 1	N = 30,323 x = 1263.5 SD = 2610.3			

USA = United States of America, x = mean, SD = standard deviation, --: not available.

**Table 4:** Moderators of COVID-19 vaccine acceptance, hesitancy and uptake rates (meta-regression and subgroup analyses).

Moderator	Number of Studies	Proportion of Outcome (95% CI)	Heterogeneity		Moderator Effect (Meta-Regression)			
					Univariate		Multivariate	
			*I*^2^ Within	*p*-Value	Coefficient	*p*-Value	Coefficient	*p*-Value
**Acceptance studies**								
**Study year**					0.03	0.75	--	--
2021	6	0.56 (0.41–0.70)	99%	<0.01				
2022	10	0.59 (0.47–0.71)	99%	<0.01				
2023	--	--	--	--				
**Region**					0.08	0.01	0.09	0.29
Africa	2	0.65 (0.52–0.76)	95%	<0.01				
Americas	8	0.47 (0.34–0.60)	99%	<0.01				
Asia	6	0.70 (0.61–0.77)	96%	<0.01				
**Slum population**					0.09	<0.01	0.12	0.03
General	13	0.56 (0.45–0.67)	99%	<0.01				
Non-general	3	0.65 (0.59–0.70)	45%	0.16				
**Sample size**					0.04	0.59	--	--
Below 1000	11	0.56 (0.44–0.68)	99%	<0.01				
Above 1000	5	0.61 (0.47–0.74)	100%	<0.01				
**Hesitancy studies**								
**Study year**					−0.03	0.84	--	--
2021	6	0.27 (0.09- 0.59)	99%	<0.01				
2022	10	0.31 (0.19–0.44)	99%	<0.01				
2023	--	--	--	--				
**Region**					−0.11	0.01	−0.13	0.13
Africa	2	0.35 (0.23–0.49)	95%	<0.01				
Americas	8	0.40 (0.23–0.61)	99%	<0.01				
Asia	6	0.17 (0.07–0.34)	99%	<0.01				
**Slum population**					−0.17	0.01	−0.21	0.17
General	13	0.32 (0.19–0.49)	99%	<0.01				
Non-general	3	0.19 (0.11–0.32)	73%	0.02				
**Sample size**					−0.13	0.22	--	--
Below 1000	11	0.35 (0.22–0.51)	99%	<0.01				
Above 1000	5	0.19 (0.06–0.44)	100%	<0.01				
**Uptake studies**								
**Study year**					0.02	<0.01	−0.06	0.04
2021	2	0.33 (0.28–0.39)	70%	0.07				
2022	3	0.14 (0.05–0.31)	95%	<0.01				
2023	1	0.44 (0.41–0.47)	--	--				
**Region**								
Africa	1	0.44 (0.41–0.47)	--	--	−0.10	<0.01	−0.13	0.31
Americas	2	0.25 (0.17–0.35)	90%	<0.01				
Asia	3	0.17 (0.05–0.44)	99%	<0.01				
**Slum population**					--	--	--	--
General	6	0.23 (0.13–0.39)	98%	<0.01				
Non-general	0	--	--	--				
**Sample size**					0.15	0.15	--	--
Below 1000	3	0.16 (0.05–0.38)	96%	<0.01				
Above 1000	3	0.33 (0.22–0.47)	99%	<0.01				

--: Not applicable, Africa included Uganda and Kenya, Americas included USA and Brazil, Asia included India, Bangladesh, and Pakistan; non-general included studies were done among parents, hospital patients and healthcare workers.

**Table 5:** Factors associated with COVID-19 vaccination and reasons for hesitancy.

Author	Country	Associates Factors	Qualitative Reasons for Hesitancy
Lennon et al., 2022 [25]	USA	Acceptance: Gender; female (−) Ethnicity; non-white (−), residence; rural (−) Education; college and post-graduate (+)	N/A
Kusuma et al., 2022 [27]	India (Delhi)	Hesitancy: Older age (+), low perceived susceptibility and severity of COVID-19 (+), low self-efficacy to protect against COVID-19 (+), awareness and use of Aarogya Setu App (−)	A belief that they had immunity; COVID-19 was a hoax; the vaccine was not necessary; did not want to disturb the natural bodily systems by the vaccine.
Hasan et al., 2022 [28]	Bangladesh	Acceptance: Older age (+), Adequate knowledge of COVID-19 (+), comorbid patients in the households (+), religious misconceptions (−), doubt on safety of the vaccine (−)	N/A
Sunil et al., 2021 [29]	India (Bengaluru)	Uptake: Young age (+), gender; males (+), religion; Christians (+), Education; graduates (+), Occupation: clerical and skilled workers (+), SES; upper middle (+).	Mild or serious adverse effects were more reported among women than men across all age groups.
Aguilar et al. 2021 [30]	Brazil	Hesitancy: Younger age (+), low perceived benefit vaccination (+) Parental acceptance: acceptance among parents themselves (+)	Concerns about vaccine efficacy, potential side effects, low incidence of COVID-19 cases and low perceived susceptibility
Cohrs et al., 2022 [31]	USA	N/A	Adverse effects and cost of COVID-19 vaccine
Nasimiyu et al., 2022 [32]	Kenya (Kibera and Asembo)	Acceptance: Education; post-secondary (−)	Safety concerns, insufficient information to decide, low-risk perception and lack of belief in vaccine
Doherty et al., 2021 [33]	USA (North Carolina)	Hesitancy: Gender; female (+), Ethnicity; Black (+), calendar month (+), safety concerns and government distrust (+)	Safety and efficacy concerns and government mistrust
Patwary et al., 2022 [34]	Bangladesh	Acceptance: being confident (+), complacent (+), calculative (+), and responsible (+) Anti-vaccine attitudes (−), Information sources; newspaper (+)	N/A
Crozier et al., 2022 [35]	USA (Alabama)	Acceptance: Gender; male (+), ethnicity; non-Hispanic (+), older age (+), residence; urban (+)	N/A
Tamysetty et al., 2021 [36]	India (Mumbai, Bengaluru, Kolkata and Delhi)	N/A	Possible side effects, the uncertainty of getting the vaccine, safety concerns, long distance to vaccination centre, and inability to spare a day from work
Qasim et al., 2022 [37]	Pakistan (Karachi)	Acceptance: Knowledge and awareness of vaccine (+), trusted sources of information (+), good health literacy (+), Occupation; healthcare (+) Negative personal beliefs (−), vaccine mistrust (−), negative public perceptions (−)	N/A
Bhartiya et al., 2021 [38]	India (Mumbai)	Acceptance: Older age (+), gender; male (+), Education; post-graduate (−), and occupation; blue collar (+)	N/A
Kazmi et al., 2022 [39]	Pakistan (Islamabad and Rawalpindi)	Uptake and acceptance: Higher education (+), being employed (+), prior infection in the family (+), family vaccination (+), knowing of and living close to a vaccination centre (+) and being worried about COVID-19 (+)	N/A
Kawuki et al., 2023 [41]	Uganda	Uptake: Older age (+) and Ethnicity/Tribe; Batooro (+), Knowledge level (+), perceived benefits (+) and cues to action (+). Depressive symptoms (−), perceived barriers; serious side effects and long distances (−), Unemployment (−), Religion; Moslem (+) and Tribe; Basoga (−)	N/A
Abedin et al., 2021 [42]	Bangladesh	Hesitancy: Older age (+), low education (+), Occupation; day-laborers (+), having chronic diseases (+), low confidence in the country’s healthcare system (+), residence: slum (+)	N/A
Wang et al., 2021 [45]	USA (Delaware)	Acceptance: Ethnicity; Black (−), COVID test history (+)	N/A
Garcini et al., 2022 [46]	USA (South Texas)	N/A	Mistrust of manufacturers and administrators, concerns about vaccine safety, fear of discrimination/stigmatisation, fear of exploitation/ manipulation by the government or health authorities, and having personal information mishandled. Being undocumented, fear-inducing myths and beliefs, limited information and logistics of vaccination access
Campagnoli et al., 2022 [47]	USA (Chicago)	Acceptance: older age (+), Ethnicity; white (−), big household size (+), and trust in healthcare workers (+)	Missed opportunity (not having access to a doctor or not seen a doctor lately), fear of short- and long-term side effects, not enough research on the COVID-19 vaccines, concerns about vaccine effectiveness, and government mistrust
Robinson et al., 2022 [48]	USA (Alaska and Idaho)	Uptake: Vaccine confidence (+), high perceived susceptibility (+), severity (+), and benefits (+), convenient access (+), availability (+) and cues to action; SMS (+)	N/A

N/A = Not available, USA = United States of America, (+) = positive association, (−) = negative association.

## Data Availability

Data supporting this systematic review are available in the reference section. In addition, the analysed data used in this systematic review are available from the author on reasonable request.

## Supplementary Materials

The following supporting information can be downloaded at: https://www.mdpi.com/article/10.3390/vaccines11050886/s1, Supplementary Table S1: Quality assessment of included studies.
