# Supplementary material for: COVID-19 Vaccine Acceptance, Attitude and Perception among Slum and Underserved Communities: A Systematic Review and Meta-Analysis
Source: Vaccines (Basel). 2023 Apr 23;11(5):886. doi: 10.3390/vaccines11050886 (PMC10222524; doi:10.3390/vaccines11050886)
Supplement: Supplementary file 1 [file vaccines-11-00886-s001.zip › vaccines-2290623-supplementary.pdf]

Supplementary Table S1A. Overall Quality assessment of included studies by Mixed Methods Appraisal Tool (MMAT) – Version 2018

**Criteria**		**QND n=19 (%)**	**QL n=3 (%)**	**MM n=2 (%)**
QND1. Is the sampling strategy relevant to address the research question?	Yes	100		
	No	0		
	Can't tell	0		
QND2. Is the sample representative of the target population?	Yes	81.8		
	No	13.6		
	Can't tell	4.6		
QND3. Are measurements appropriate (clear origin, or validity known, or standard instrument)?	Yes	95.5		
	No	0		
	Can't tell	4.5		
QND4. Is the risk of non-response bias low?	Yes	45.5		
	No	0		
	Can't tell	54.5		
QNR5. Is the statistical analysis appropriate to answer the research question?	Yes	86.4		
	No	13.6		
	Can't tell	0		
QL1. Is the qualitative approach appropriate to answer the research question?	Yes		100	
	No		0	
	Can't tell		0	
QL2. Are the qualitative data collection methods adequate to address the research question?	Yes		100	
	No		0	
	Can't tell		0	
QL3. Are the findings adequately derived from the data?	Yes		100	
	No		0	
	Can't tell		0	
QL4. Is the interpretation of results sufficiently substantiated by data?	Yes		100	
	No		0	
	Can't tell		0	
QL5. Is there coherence between qualitative data sources, collection, analysis and interpretation?	Yes		100	
	No		0	
	Can't tell		0	
MM1. Is there an adequate rationale for using a mixed methods design to address the research question?	Yes			100
	No			0

	Can’t tell	0
MM2. Are the different components of the study effectively integrated to answer the research question?	Yes	100
	No	0
	Can’t tell	0
MM3. Are the outputs of the integration of qualitative and quantitative components adequately interpreted?	Yes	50
	No	0
	Can’t tell	50
MM4. Are divergences and inconsistencies between quantitative and qualitative results adequately addressed?	Yes	100
	No	0
	Can’t tell	0
MM5. Do the different components of the study adhere to the quality criteria of each tradition of the methods involved?	Yes	100
	No	0
	Can’t tell	0

QND: quantitative descriptive studies; QL: qualitative studies; MM: mixed-method studies

The MMAT assesses the quality of qualitative, quantitative, and mixed methods studies. It focuses on methodological criteria and includes five core quality criteria for each of the following five categories of study designs: (a) qualitative, (b) randomized controlled, (c) non-randomized, (d) quantitative descriptive, and (e) mixed methods. The tool uses three response options to rate each article: 'Yes' meaning the criterion is met, 'No' meaning the criterion is not met, and 'Can't tell' when there is not enough information in the paper to judge if the criterion is met or not. After rating all the studies in each study design, a summary in terms of percentage is given for each of the three response options.

Supplementary Table S1B. Quality assessment of each study by Mixed Methods Appraisal Tool (MMAT) – Version 2018

**Author**	**Assessment 1**	**Assessment 2**	**Assessment 3**	**Assessment 4**	**Assessment 5**	**Overall Bias**
**Quantitative studies**						
Lennon et al. [25]	Yes	Yes	Yes	Can't tell	Yes	Low
Kusuma et al. [27]	Yes	Yes	Yes	Yes	Yes	Low
Hasan et al. [28]	Yes	Yes	Yes	Can't tell	Yes	Low
Sunil et al. [29]	Yes	Yes	Yes	Can't tell	No	High
Aguilar et al. [30]	Yes	Yes	Yes	Can't tell	Yes	Low
Cohrs et al. [31]	Yes	No	Yes	Yes	Yes	Low

Nasimiyu et al. [32]	Yes	Yes	Yes	Yes	Yes	Low
Doherty et al. [33]	Yes	Yes	Yes	Yes	Yes	Low
Patwary et al. [34]	Yes	Yes	Yes	Yes	Yes	Low
Crozier et al. [35]	Yes	Yes	Yes	Can't tell	Yes	Low
Bhartiya et al. [38]	Yes	Yes	Yes	Can't tell	No	High
Kazmi et al. [39]	Yes	Yes	Yes	Can't tell	Yes	Low
Coman et al. [40]	Yes	No	Yes	Can't tell	Yes	High
Kawuki et al. [41]	Yes	Yes	Yes	Yes	Yes	Low
Abedin et al. [42]	Yes	Can't tell	Yes	Yes	Yes	Low
Nabirye et al. [43]	Yes	Yes	Yes	Can't tell	Yes	Low
Mamun et al. [44]	Yes	Yes	Can't tell	Can't tell	No	High
Wang et al. [45]	Yes	Yes	Yes	Yes	Yes	Low
Campagnoli et al. [47]	Yes	No	Yes	Yes	Yes	Low
**Qualitative studies**						
Alam et al. [26]	Yes	Yes	Yes	Yes	Yes	Low
Robinson et al. [48]	Yes	Yes	Yes	Yes	Yes	Low
Qasim et al. [37]	Yes	Yes	Yes	Yes	Yes	Low
**Mixed-method studies**						
Tamysetty et al. [36]	Yes	Yes	Yes	Yes	Yes	Low
Garcini et al. [46]	Yes	Yes	Can't tell	Yes	Yes	Low

Assessments 1-5 correspond to the specific five assessment questions for quantitative, qualitative and mixed-method studies detailed in Supplementary Table S1A
